# Lecture on “Pericementitis” before the Dental Association of Northern New Jersey

**Published:** 1884-09

**Authors:** Frank Abbott


### Independent Practitioner.

Vol. V. September, 1884. No. 9.

### Original Communications.

### LECTURE ON "PERICEMENTITIS" BEFORE THE CENTRAL DENTAL ASSOCIATION OF NORTHERN NEW JERSEY.

BY FRANK ABBOTT, M. D.

*Mr. President and Gentlemen:*

I suppose you are all aware that at this season of the year, particularly, we are all very busy, consequently it has been impossible for me to write out what I expect to say to you.

Pericementitis, the subject upon which I am to speak this evening, is so often met with, and sometimes so very difficult to treat, that it becomes a matter of great importance to us as to what will assist in removing or allaying the disturbance. It requires, on the part of the practitioner, a full knowledge of pathology and therapeutics in order to diagnosticate and treat it successfully. It is inflammation of the pericementum (periosteum) which surrounds the root of the tooth, as it stands in the alveolus. There is probably no disease which gives so much annoyance to the dentist, and to the patient so much trouble, as this. We are asked, "Cannot something be done to relieve this severe soreness of my teeth?" Not only is the patient in a condition of pain and distress, but the teeth are sore to the touch; he is unable to masticate upon them with any degree of comfort. Chronic pericementitis is a condition that may be present, it is true, and the patient really not be aware of it, nor can it be detected by percussion with an instrument upon the tooth, nor by forcing it back and forth with the thumb and finger, still some such remote organ as the eye or ear may be effected. It is not an uncommon thing for a persistent facial neuralgia to develop, which may baffle the skill of the most astute of general practitioners of medicine, or an affection of the eye or ear which does not yield to treatment at the hands of oculists or aurists.

In order that we may understand more clearly this pathological condition, it perhaps would be well for us first to comprehend the tooth physiologically It is necessary for us to know how the tooth is built up, in order to understand the disease, or the peculiar conditions, which we call pericementitis.

The pulp originally occupied the entire portion of the tooth now dentine. By the process of calcification the row of odontoblasts that was around the outer edge of this pulp soon became filled with lime-salts, and was converted into what I term dentine cells; immediately inside of these formed dentine cells another row of odontoblasts is arranged, and so on really as long as the tooth remains in the jaw and is alive. The crown of the tooth is first formed, and subsequently the root.

In the formation of dentine, of course, the reticulum, which was living, still remains alive after the lime-salts are deposited. The dentinal fiber stops before reaching the enamel, and is either lost in a granular mass, or divided into two or three branches, in order to accommodate itself to a proper supply of the finer structure of the enamel. The enamel prisms are undoubtedly pierced with very minute fibers of living matter, as shown in the diagram, but as yet they have not been positively identified.

This portion of the tooth has, of course, a covering which is called enamel, and is formed by what is known as the enamel organ. Extending over the entire crown is an epithelial covering known as "Nasmyth's membrane." This thin membrane, by the friction of mastication and cleansing, soon becomes worn off, and the finely polished enamel is brought to view. This portion of the tooth is nourished entirely from the pulp, not only the dentine, but the enamel as well. When we come to the formation of the root, the enamel and the crown portion of the dentine having previously been formed, another element enters into the construction, viz., the cementum. This, in its construction, is the same as ordinary compact bone. It is the product of the pericementum, is attached to it and nourished by it.

The best analysis we have of the tooth is that the enamel possesses 3½ parts of organic material, and 96½ parts inorganic. In the dentine we have 28 parts of organic and 72 of inorganic; and in the cementum again we have 33 parts of organic and 67 inorganic. I give these figures, not claiming that they are always the same. Undoubtedly we would arrive at a different analysis in each instance, finding a varying amount of the different materials of which a tooth is composed. The arrangement of the fibers of connective tissue of the pericementum is an interesting feature to the dental student. If you will examine this diagram carefully, you will observe that the ends of these fibers attached to the socket are toward the gum, while the ends attached to the root point toward its end, so that when the teeth are bitten upon, the fibers stretch and allow of a slight movement of the tooth. When the pressure is removed they contract again, and the tooth assumes its suspended position. Were the teeth placed in the jaws solidly, many more would be broken than now are. In enumerating the different causes of pericementitis, I presume I shall omit many, but I will endeavor to give the principal ones. They are as follows: Any organic disturbance of the dentine or cement; direct irritation of the pericementum; putrefaction of the dead pulp in the canal, or any portion that is left when extirpation has been attempted; putrefaction of the organic portion of the dentine; mercurial poisoning; uterine irritation during gestation or the menstrual period, and exostosis.

The usual organic disturbance of the dentine or cement usually involves caries. We remove this and seal the cavity, and thus prevent further irritation of the living portion of the organ, and generally no further trouble is experienced.

It is often the case, however, that the living tooth, when the neck becomes denuded, will cause the patient severe neuralgic pains and slight pericementitis. This may be relieved by the use of an alkaline solution, such as bi-carbonate of soda in water.

When the tooth is decayed to the pulp, we have direct irritation of that organ. The common practice is to cap such an exposed pulp. I am afraid, however, that it is not as common as it ought to be. From what I see and hear, I fear that a great many men are too reckless in sacrificing the life of the pulps of teeth. Its death is productive of so much trouble and distress, so many alveolar abscesses, so many sleepless nights, and sometimes even the death of the patient, that it becomes a matter of great importance to us and to our patients. Besides, the preservation of the natural color of the tooth is of paramount importance, particularly if the tooth be in the anterior portion of the mouth. That bluish-grey or greyish-brown color which a pulpless tooth assumes, is anything but pleasant to look upon. This discoloration is indicative of the putrefaction of the organic portion of the dentine, to which I shall refer further on. That the operation of capping the exposed pulp of a tooth is not always attended with the desired result, I am well aware; but should the first attempt fail, the capping material should be removed and another trial made; yes, two, and even more, provided the patient will submit to the long-continued experimentation. If a portion of the pulp unfortunately dies, it should be amputated, and the remainder preserved, if possible, as a portion of live pulp in a tooth will preserve its natural color. I am aware that this kind of work is very trying to the nerves of the dentist as well as the patient, and perhaps it is not to be wondered at that sometimes endurance yields, and the pulp is devitalized and its removal attempted so frequently.

I had a patient to-day, whose left upper central incisor was pulpless, and had assumed the characteristic grey color. The right central had some months since exhibited signs of chronic pericementitis, but its color was good. Upon removing an imperfect filling which it contained, I found that the carious process had penetrated to the pulp, and a portion of it was dead. I removed that portion and made a soothing application to the remaining live portion, in the hope of preserving it.

The preservation of exposed pulps I consider one of the greatest achievements in modern dental surgery.

PUTREFACTIVE IRRITATION.

The general impression is that the cause of all the distress which we call pericementitis is due to the putrefaction of the dead pulp when left in a tooth. But how shall we account for this condition when it occurs after the treatment of such teeth by men who are positive that they have removed every particle of dead tissue? The fact is, that in the hands of our most careful dentists a portion of dead pulp is left in the canals of about one-half of the cases treated. For instance, how much credit would you put in the statement of any man who would tell you that he could, with certainty, always remove the entire pulp from the first bicuspid, or either of the molars in the upper jaw, or from either of the molars in the lower jaw?

It matters very little whether you leave the pulp entire, or a piece as large as the point of a needle in the canal; if that putrefies there is danger, although the initial point is not as threatening. Even though the pulp is removed from the tooth entire, and the root is sealed up as tightly as possible, there is still twenty-eight per cent. of the dentine dead and undergoing slight putrefaction, unless every particle of air is excluded, which is seldom, if ever, done. Now if the patient takes a severe cold by sitting in a draft, or should bite upon such a tooth a little severely at any time, producing sufficient irritation to cause a determination of blood to the part, is it at all unreasonable to suppose that the living portion of the cementum, coming as it does in direct contact with this putrefying portion of the dentine, will absorb an excessive amount of it, producing an irritation which is directly conveyed to the pericementum? I have no doubt that thousands of cases of pericementitis which have puzzled the brain of many a good and faithful dental surgeon may be accounted for in this manner.

Many methods are resorted to, and many materials used to relieve this painful condition, among which may be mentioned counter-irritants, such as a small mustard or capsicum plaster, the application of leeches, of cantharidal collodion, aconite and iodine, or a piece of hot fig or raisin. I have obtained more immediate and lasting relief from the use of the two latter than from any other remedies I have ever tried. The aconite used in this mixture is a saturated tincture, and made by only one man, so far as I know—T. J. MacMahan, 142 Sixth Avenue, New York. This, mixed with an equal amount of tr. iodine, is used. It may be painted on the gum over the affected tooth, every three hours, until the inflammation is checked. Recently, however, I have been in the habit of recommending the heating of the inside of a raisin from which the seeds have been removed, as hot as it can be borne, and placing it on the gum over the root, and repeating this every half hour. In this manner I have reduced the inflammation in many very obstinate cases. I have used the aconite and iodine for fifteen years or more, it very seldom failing to give relief if applied before suppuration takes place.

DIRECT IRRITATION OF THE PERICEMENTUM ITSELF.

This takes place in several different ways. Perhaps the most prolific cause, however, is that of the collection of tartar around the roots under the gums. That brings me to a point which is very interesting. It is a question you have, doubtless, asked yourselves dozens of times: Where does this calcareous deposit come from? You all very well know that the function of the pericementum (periosteum) under irritation is to secrete and deposit lime-salts; for example, in the repair of fracture, and the reproduction of bone. As soon as the slightest deposit takes place under the free margin of the gum, or an inflammation of the gum from any cause deprives the part of its proper nourishment, the tissue becomes detached from the neck of the tooth, thus opening a pocket for the reception of foreign substances, which may come in contact with the pericementum and produce an irritation which shall cause a deposition or outpouring of lime-salts, and these readily attach themselves to the irregular surface of the cementum. This is the condition when that excessive ulceration takes place that is known as "pyorrhea alveolaris."

This is not an original idea of mine, but should be credited, I believe, to Dr. Niles, of Boston.

Now, the removal of this material from around the root of the tooth is in many instances easy, and all that is necessary to relieve the pericemental disturbance. Like many of my brother practitioners, I was in the habit, until within the past two years, of using antiseptics, stimulants and astringents in all such cases, but for the last-named period I have depended in almost every instance upon the thorough removal of all deposit from the roots, and trust nature to restore the parts to health. I prescribe for the patient a saturated solution of chlorate of potash in cold water, and direct him to rinse the mouth with it every hour, if a severely inflamed case; three or four times a day if the attack be mild. The success I meet with in this treatment warrants me in recommending its trial.

### TRAUMATIC INJURIES.

We produce pericementitis by moving teeth about for the purpose of obtaining room to fill, and also from long and too severe malleting upon dead teeth. In very large operations it would perhaps be well to adopt the new mode of putting in filling by the Herbst method; rubbing them in with a small burnisher. In many cases where large operations are to be performed in molar teeth, it is advisable to use amalgam or some cement, rather than risk hard and continued labor upon gold fillings. Eisfelder's cement, a German preparation, is the most perfect and hardest cement for the teeth I have ever used. You may not be able to get it here, but can order it fiom Germany. Some of these cements may be used to advantage, rather than gold filling, in discolored teeth, or where very much of the crown of a molar or bicuspid has been broken away. By placing a small layer of gutta percha at the cervical portion, large approximate cavities may be successfully filled with it.

### MERCURIAL POISONING.

Salivation very frequently, if not always, produces pericementitis. The organic principle of saliva (ptyaline) acts as a poison to the mucous membrane of the mouth, when the saliva is secreted excessively. This produces an inflammation of the gum around the teeth, by which its proper nourishment is cut off, and its detachment from the necks of the teeth is the consequence. This inflammation extends to the pericementum entire, and the loosening, and perhaps loss, of the teeth is the result. This is often the beginning of pyorrhea alveolaris.

In mercurial poisoning, the treatment consists in keeping the teeth as clean as possible, and rinsing the mouth every fifteen minutes or half-hour with a solution of chlorate of potash in cold water, and touching the gums with tincture of iodine, provided the inflammation does not subside readily. I believe this to be ample treatment to relieve disturbances of this kind.

### FUNCTIONAL IRRITATION.

Uterine irritation during gestation or the menstrual period may produce pericementitis, either in a living or so-called dead tooth. A living tooth may be carious, or denuded of its natural covering at the neck. It is no uncommon thing for ladies to suffer severely at such times; as they recover from that the pericementitis subsides. When treatment is, however, demanded, the painting of the gum once with the aconite and iodine preparation will usually be sufficient to relieve the patient, provided the tooth is a pulpless one. This relief may be permanent, or the inflammation may return again within twenty-four hours, a week, or at any subsequent time. If the teeth be alive and carious, fill with gutta percha temporarily. If the necks of the teeth are exposed and irritated, use the soda solution.

### HYPERTROPHIES.

Exostosis is known as a condition of formative irritability, i. e., the pericementum is in a low, chronic state of inflammation, which results in an increase in size or thickness. As the cementum increases in thickness the surrounding alveolus must of necessity be absorbed. When pericementitis is developed it is in consequence of this absorption going on more slowly than the tumor grows. Pressure upon the pericementum, and consequent inflammation, is the result. When a diagnosis of exostosis is made, the only relief consists in extracting the tooth.

